# Registro REBECGA: Um Marco no Cuidado de Gestantes com Doença Cardíaca no Brasil

**DOI:** 10.36660/abc.20250370

**Published:** 2025-08-20

**Authors:** Imara Correia de Queiroz Barbosa, Gláucia Maria Moraes de Oliveira

**Affiliations:** 1 Universidade Federal de Campina Grande Campina Grande PB Brasil Universidade Federal de Campina Grande, Campina Grande, PB – Brasil; 2 Universidade Federal do Rio de Janeiro Rio de Janeiro RJ Brasil Universidade Federal do Rio de Janeiro, Rio de Janeiro, RJ – Brasil

**Keywords:** Gravidez, Doenças Cardiovasculares, Mortalidade Materna, Cardiopatias Congênitas

A gestação é um evento marcante na vida da maioria das mulheres. Estima-se que entre 1% e 4% das gestantes apresentem alguma forma de cardiopatia congênita ou adquirida, principal causa não obstétrica de mortalidade materna em todo o mundo.^1 - 3^ Embora muitas mulheres com cardiopatia tolerem as alterações hemodinâmicas da gestação, outras podem enfrentar riscos imediatos ou tardios significativos, como sobrecarga de volume, arritmias, disfunção cardíaca progressiva e até óbito.^3 , 4^

A prevalência de doenças cardíacas na gestação vem aumentando, impulsionada por dois principais fatores: os avanços no tratamento das cardiopatias congênitas (CC), que permitem que mais crianças sobrevivam até a vida adulta e considerem a gestação, e a tendência ao adiamento da maternidade.^1 , 3 , 5^ Com o aumento da idade materna, cresce também a prevalência de comorbidades como hipertensão, diabetes e hipercolesterolemia, contribuindo para uma maior incidência de cardiopatias adquiridas complicando a gravidez.^1 , 3^

Nos Estados Unidos, a CC representa o tipo de cardiopatia mais frequente entre gestantes.^1 , 2 , 5 , 6^ Nos países em desenvolvimento, a cardiopatia reumática é a principal causa de doença cardíaca na gestação.^1 , 2 , 5^

Atualmente, existem três principais modelos de risco ou classificação utilizados para prever eventos cardiovasculares adversos na gestação em mulheres com doença cardíaca prévia: os escores CARPREG II e ZAHARA, e a Classificação da Organização Mundial da Saúde modificada (mOMS).^2 , 5 , 7^ A classificação mOMS é a mais acurada.^6^ Ela divide as doenças cardíacas por grau crescente de gravidade, do risco I ao risco IV. As pacientes incluídas no risco IV devem ser desaconselhadas a engravidar.^3^

A mortalidade materna é elevada em gestantes com hipertensão arterial pulmonar e está associada a um maior risco de parto prematuro, óbito fetal ou neonatal e baixo peso ao nascer.^8^ Mulheres com próteses valvares enfrentam baixas taxas de sucesso gestacional, pois tanto as biológicas quanto as mecânicas acarretam riscos maternos e fetais relevantes. Gestações futuras ainda geram preocupações quanto à disfunção estrutural das próteses e ao manejo complexo da anticoagulação.^9^

**Figura 1:**
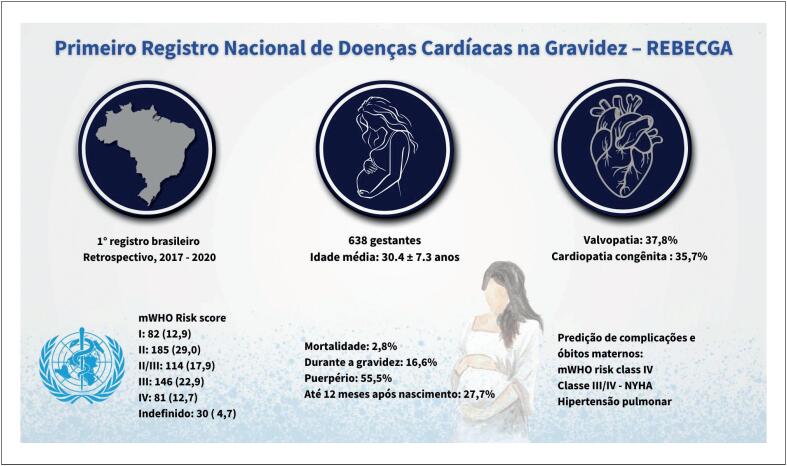
Primeiro Registro Nacional sobre Doença Cardíaca na Gestação – REBECGA. mOMS: classificação da Organização Mundial da Saúde modificada para risco cardiovascular materno; NYHA: New York Heart Association.

Nesta edição dos Arquivos Brasileiros de Cardiologia, Ávila et al. apresentam um panorama realista das doenças cardíacas na gestação no Brasil por meio da fase retrospectiva do Registro Brasileiro de Doença Cardíaca e Gravidez (REBECGA), o primeiro registro nacional deste tipo ( Figura 1 ). Ao oferecer dados diretamente relevantes à população brasileira, esta iniciativa preenche uma lacuna essencial não contemplada pelos escores internacionais de risco.^10^

Foram avaliadas 638 gestantes com diagnóstico prévio de cardiopatia entre 2017 e 2020, em seis centros brasileiros, com seguimento de até 12 meses após o parto. Embora os riscos relacionados à gestação tenham sido explicados para 64,9% das participantes, apenas um terço havia planejado a gestação. As valvopatias, predominantemente de etiologia reumática, foram o diagnóstico mais prevalente, seguidas pelas cardiopatias congênitas. O registro incluiu tanto casos estáveis quanto avançados: na avaliação inicial, 36,1% foram classificadas como alto risco (classes III/IV da mOMS), 36,5% apresentavam fatores complicadores e 29,2% tinham histórico de eventos cardíacos prévios à gestação. Foram observadas altas taxas de complicações durante a gestação e nos 12 meses subsequentes ao parto, com mais da metade dos óbitos maternos ocorrendo no puerpério.^10^

A análise preditiva das complicações cardíacas e da mortalidade materna identificou a classificação de risco mOMS e a presença de hipertensão pulmonar como covariáveis independentes associadas ao risco de complicações e/ou morte. A doença arterial coronariana foi o principal preditor de complicações obstétricas e fetais, tanto na análise univariada quanto na multivariada, com mais da metade das mulheres afetadas apresentando intercorrências. Este subgrupo se caracterizou por idade materna mais elevada, aspecto enfatizado pelo estudo REBECGA.^10^

Quanto à avaliação das gestantes com próteses valvares, 74% possuíam próteses biológicas (PB), prática amplamente adotada pela maioria dos centros de cirurgia cardíaca do país para mulheres em idade fértil, em consonância com diretrizes que buscam evitar a anticoagulação. Contudo, o estudo revelou associação entre disfunção protética – tanto biológica quanto mecânica – e complicações maternas e mortalidade, sugerindo que as PB não garantem plena segurança gestacional.^10^

Este estudo também chama atenção para a alta taxa de partos cesáreos, condizente com os dados nacionais brasileiros, que são cinco vezes superiores aos recomendados pela Organização Mundial da Saúde.^11^

É importante reconhecer as limitações inerentes a um estudo retrospectivo; ainda assim, o monitoramento centralizado dos dados garantiu consistência e qualidade entre os centros participantes.

Este trabalho é fundamental para o aprimoramento do planejamento reprodutivo entre mulheres brasileiras com cardiopatia. O planejamento familiar eficaz é essencial para uma estratificação de risco acurada relacionada à gestação e para a escolha do método contraceptivo mais adequado. O aconselhamento pré-gestacional permite uma avaliação individualizada do risco, com discussão detalhada dos riscos maternos ao longo da gestação, do parto e do puerpério, além da análise de medicamentos potencialmente teratogênicos e a busca por terapias alternativas mais seguras.^4^

O REBECGA lança as bases para futuros avanços no cuidado cardiovascular de gestantes no Brasil e abre novos caminhos para uma maternidade mais segura e melhores desfechos cardiovasculares.
