# Supplementary material for: Registro REBECGA: Um Marco no Cuidado de Gestantes com Doença Cardíaca no Brasil
Source: Arq Bras Cardiol. 2025 Aug 20;122(8):e20250370. [Article in Portuguese] doi: 10.36660/abc.20250370 (PMC12671608; doi:10.36660/abc.20250370)
Supplement: Translation

# Translation: The REBECGA Registry: A Landmark in the Care of Pregnant Women with Heart Disease in Brazil

**DOI:** 10.36660/abc.20250370i

**Authors:** Imara Correia de Queiroz Barbosa, Gláucia Maria Moraes de Oliveira

**Affiliations:** 1 Campina Grande PB Brasil Universidade Federal de Campina Grande, Campina Grande, PB – Brasil; 2 Rio de Janeiro RJ Brasil Universidade Federal do Rio de Janeiro, Rio de Janeiro, RJ – Brasil

**Keywords:** Pregnancy, Cardiovascular Diseases, Maternal Mortality, Congenital Heart Defects

Pregnancy is a major life event for almost every woman. It has been estimated that 1-4% of pregnant women have some form of congenital or acquired cardiac disease, the main non-obstetric cause of maternal mortality worldwide.^1 - 3^Although many women with heart disease tolerate the hemodynamic changes of pregnancy, others may face significant immediate or late risks of pregnancy, including volume overload, arrhythmias, progressive cardiac dysfunction, and death.^3 , 4^

There is a growing prevalence of heart disease during pregnancy, driven by two main factors: advances in the treatment of congenital heart disease (CHD), which have allowed more children to survive into adulthood and consider pregnancy, and the trend of delayed childbearing.^1 , 3 , 5^As maternal age increases, so does the prevalence of comorbidities such as hypertension, diabetes, and hypercholesterolemia, contributing to a higher incidence of acquired heart disease complicating pregnancy.^1 , 3^

In the United States, CHD represents the most frequent cardiac disorder among pregnant women.^1 , 2 , 5 , 6^In developing countries, rheumatic heart disease is the leading cause of cardiac disease in pregnancy.^1 , 2 , 5^

There are currently three primary risk or classification models used to predict adverse cardiovascular events in pregnancy for women with pre-existing cardiac disease. These are the CARPREG II risk score, the ZAHARA risk score, and the modified World Health Organization classification of maternal cardiovascular risk (mWHO).^2 , 5 , 7^The mWHO classification is the most accurate.^6^This classification divides heart diseases by increasing level of severity from risk I to risk IV. Patients included in the IV-WHO risk should be advised against pregnancy.^3^

Maternal mortality is high in pregnant women with pulmonary arterial hypertension and is associated with an increased risk of preterm birth, fetal or neonatal death, and low birth weight.^8^Women with valve prostheses face low rates of successful pregnancy, as both biological and mechanical prostheses carry significant maternal and fetal risks. Future pregnancies are associated with concerns such as structural prostheses dysfunction and the complex management of anticoagulation.^9^

In this issue of the *Arquivos Brasileiros de Cardiologia* , Ávila et al. present a realistic overview of heart disease in pregnancy in Brazil through the retrospective phase of the Brazilian Registry of Heart Disease and Pregnancy (REBECGA), the first national registry of its kind ( Figure 1 ). By offering insights directly relevant to the Brazilian population, this initiative fills a crucial gap not covered by international risk scores.^10^

**Figure 1:** First National Registry on Heart Disease in Pregnancy – REBECGA. mWHO: modified World Health Organization classification of maternal cardiovascular risk, NYHA: New York Heart Association.

A total of 638 pregnant women with a prior diagnosis of heart disease were evaluated between 2017 and 2020 across six centers in Brazil, with follow-up extending up to 12 months postpartum. Although pregnancy-related risks were explained to 64.9% of the participants, only one-third had planned their pregnancies. Valvular heart disease, predominantly of rheumatic etiology, was the most prevalent diagnosis, followed by CHD. The registry included both stable and advanced cases: at the initial evaluation, 36.1% were classified as high risk (mWHO risk class III/IV), 36.5% had complicating factors, and 29.2% had a history of cardiac events before pregnancy. The study documented high rates of complications during pregnancy and within the 12-month postpartum period, with over half of maternal deaths occurring in the puerperium.^10^

The prediction analysis for cardiac complications and maternal mortality identified the mWHO risk classification and the presence of pulmonary hypertension as independent covariates associated with the risk of cardiac complications and/or death. Ischemic heart disease was the most significant predictor of obstetric and fetal complications in both univariate and multivariate analyses, with more than half of the affected pregnant women experiencing complications. This subgroup was characterized by a higher maternal age, a point emphasized by the REBECGA study.^10^

Regarding the evaluation of pregnant women with prosthetic heart valves, 74% had biological prostheses (BP), a practice widely adopted by most cardiac surgery centers in Brazil for women of childbearing age, following valvular heart disease guidelines aimed at avoiding lifelong anticoagulation. However, the current study revealed an association between prosthetic valve dysfunction, both biological and mechanical, and maternal complications and mortality, suggesting that BP does not fully ensure pregnancy safety.^10^

This study draws attention to the high rate of cesarean deliveries, consistent with Brazilian national data, which is five times higher than the rates recommended by the World Health Organization.^11^

It is important to acknowledge the limitations inherent in a retrospective study; nonetheless, centralized data oversight helped ensure consistency and quality across participating centers.

This study is crucial for improving reproductive planning among Brazilian women with heart disease. Effective family planning is vital for accurate risk stratification related to pregnancy and for selecting appropriate contraceptive methods. Pre-pregnancy counseling enables individualized risk assessment, encompassing a thorough discussion of maternal risks throughout pregnancy, delivery, and the postpartum period, as well as the evaluation of potentially teratogenic medications and the consideration of safer alternative therapies.^4^

REBECGA lays a foundation for future improvements in cardiovascular care for pregnant women in Brazil and opens new paths toward safer motherhood and better cardiovascular outcomes.
